# Supplementary figures and images for: Full-length transcriptome and targeted metabolome analyses provide insights into defense mechanisms of Malus sieversii against Agrilus mali
Source: PeerJ. 2020 May 14;8:e8992. doi: 10.7717/peerj.8992 (PMC7231508; doi:10.7717/peerj.8992)

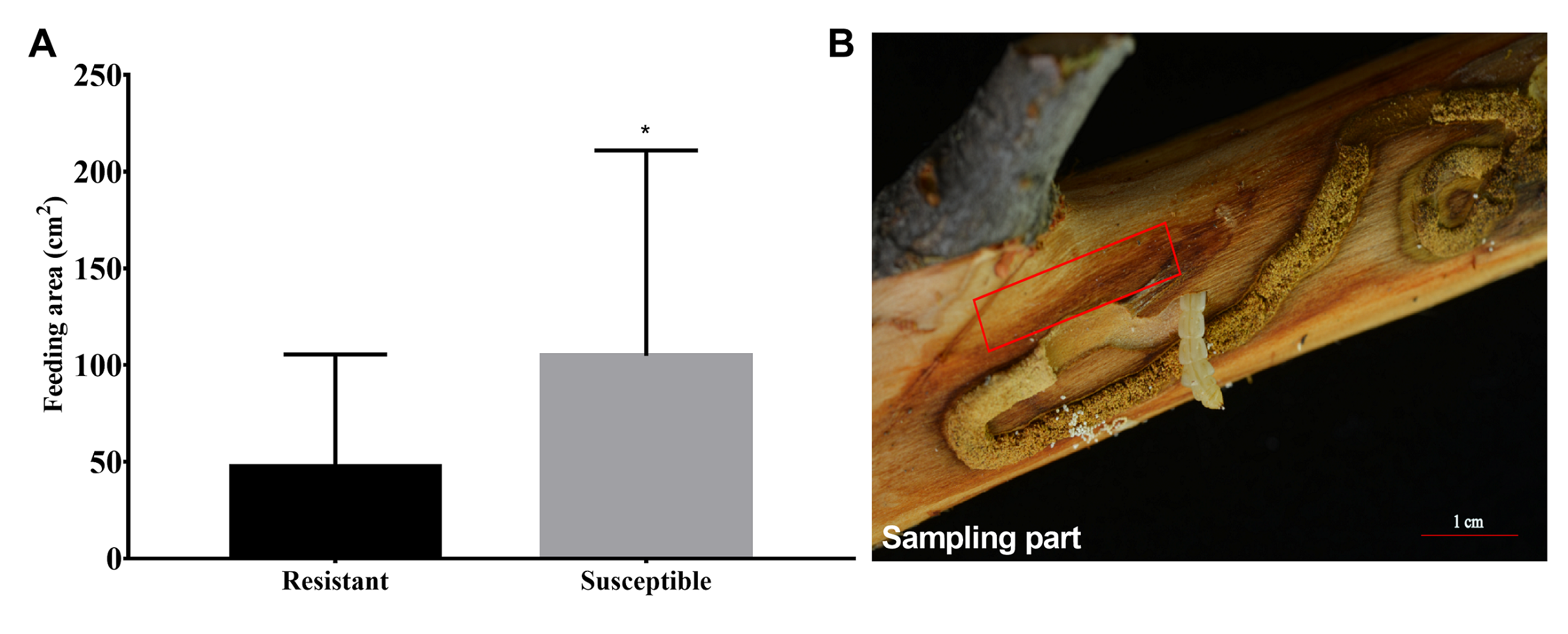

Supplement: Figure S1 — (A) The average size of infected area in the leaves of resistant and susceptible strains. Put moisture-proof mud into a 30 × 50 cm plastic box, insert 10 resistant shoots and 10 susceptible shoots into the moisture-proof mud, then put 30 insect adults into each box and record the size of the holes on the leaves eaten by insects every day . In this experiment, each shoot was placed in each box and each shoot had 8 fresh leaves and had a a perimeter of 1–2 cm. (B) A 0.5 × 2 cm phloem near the site of obvious infection was selected as a sample. [file peerj-08-8992-s001.png]

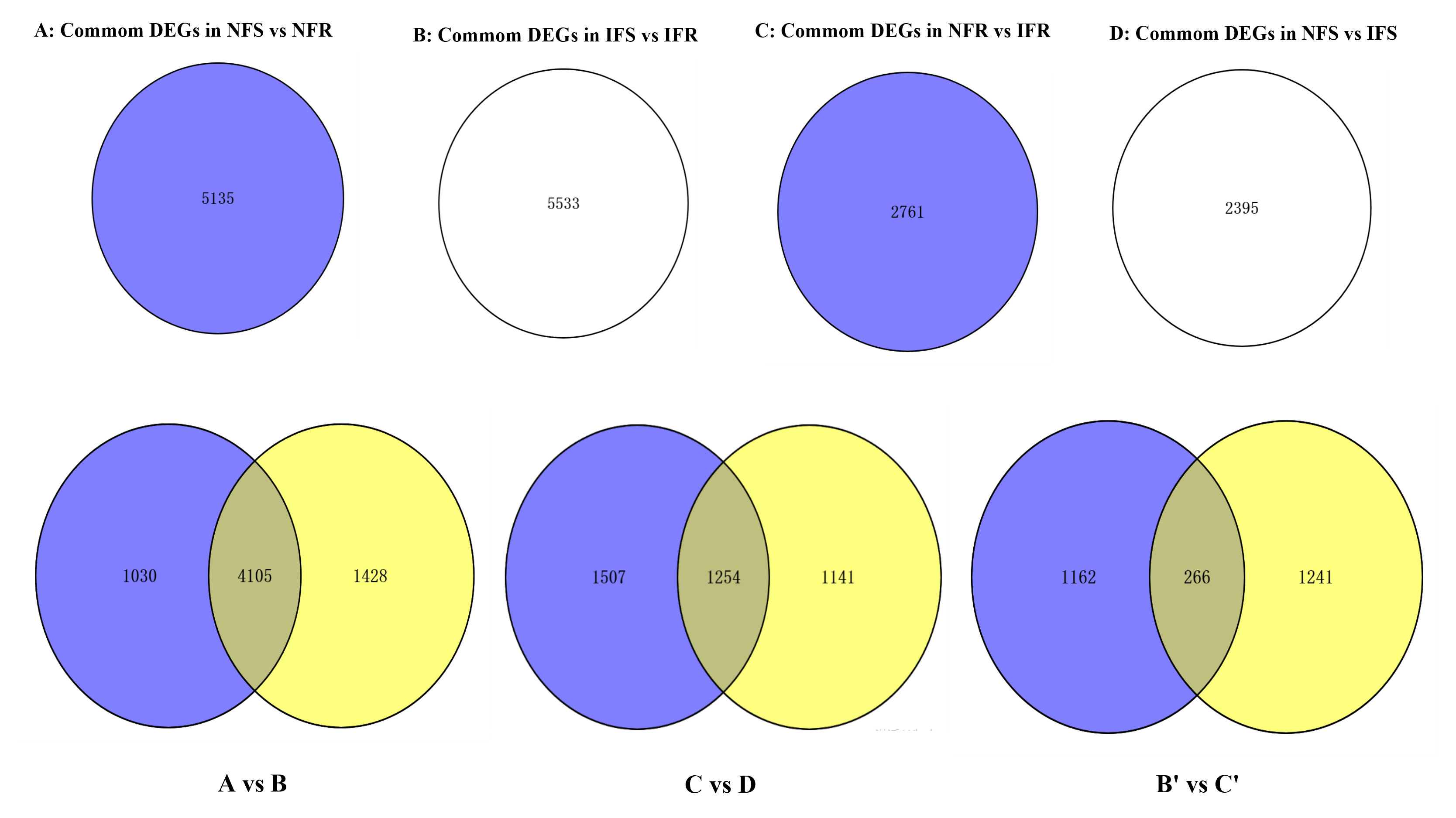

Supplement: Figure S2 — B’ is all insect response genes (1428) in resistant and susceptible plants after infection, C’ is all insect response genes (1507) in resistant plants. [file peerj-08-8992-s002.png]

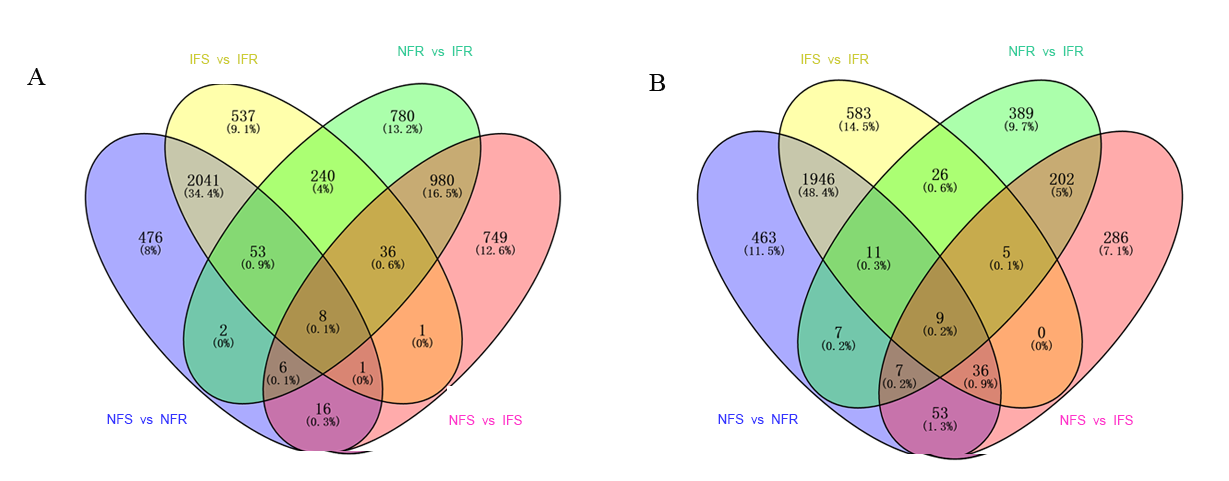

Supplement: Figure S3 — (A) Up-regulating DEGs. (B) down-regulating DEGs. [file peerj-08-8992-s003.png]

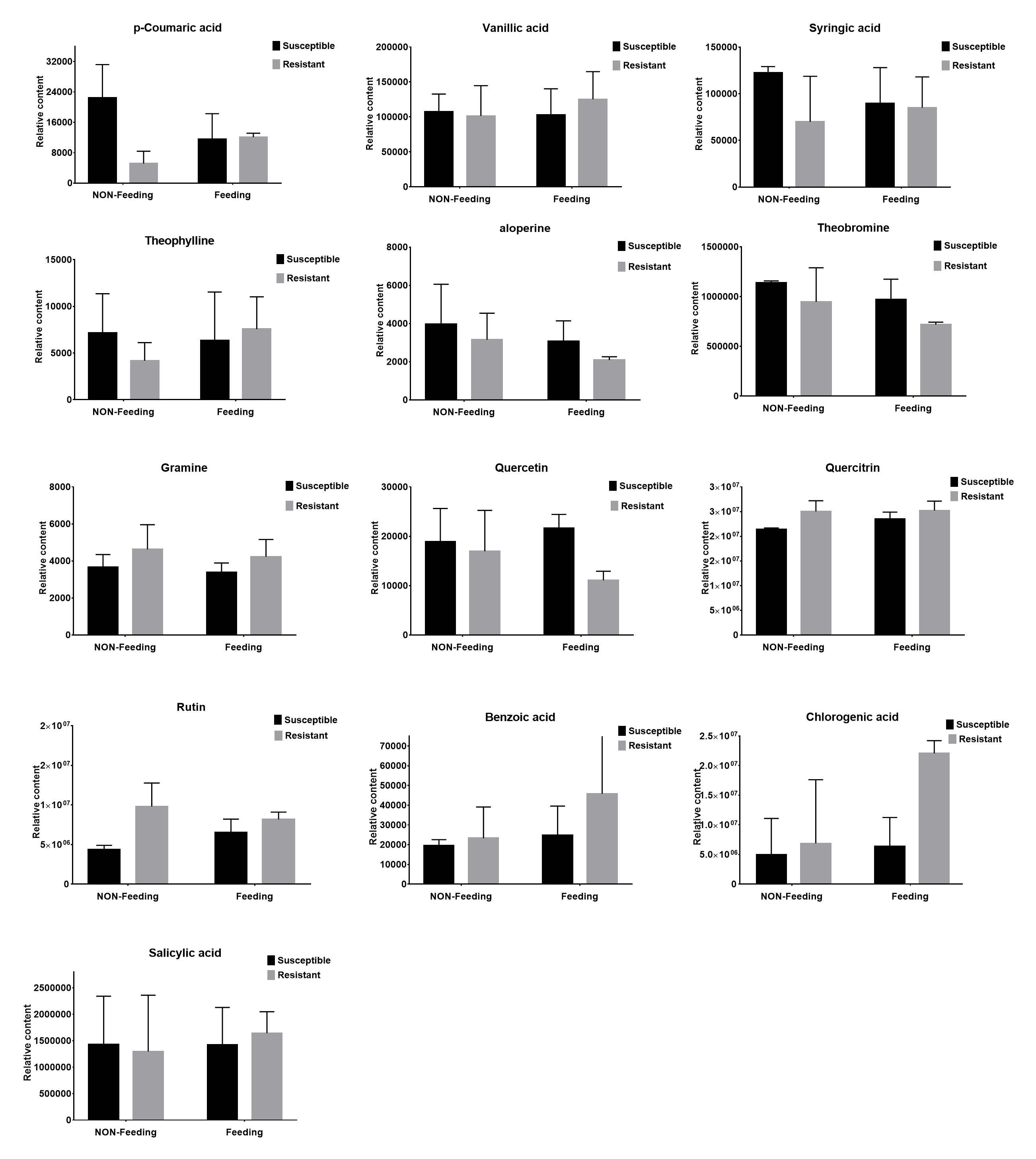

Supplement: Figure S4 — Non-Feeding represents uninfested samples, Feeding represents infested samples. [file peerj-08-8992-s004.png]
