# Supplementary material for: Full-length transcriptome and targeted metabolome analyses provide insights into defense mechanisms of Malus sieversii against Agrilus mali
Source: PeerJ. 2020 May 14;8:e8992. doi: 10.7717/peerj.8992 (PMC7231508; doi:10.7717/peerj.8992)
Supplement: Table S1 [file peerj-08-8992-s005.docx]

|  | | | |
| --- | --- | --- | --- |
| ID | Gene name | Forward | Reverse |
| F01.PB7083 | *TIFY9* | CAAGGTGGAGCAAGTCCTGT | ATGCTATCCGCCTTGTCGAG |
| F01.PB16606 | *TCM4* | CAAAACTCCGCGGCAAGAAA | GCGAGGTCGGAGAGATTACG |
| F01.PB6941 | *BAC7* | TTTCACAAGGCCATCTCGCT | GGGCGAAACAGCTGAATGTG |
| F01.PB49846 | *EDR1* | GAACTCCGGAATGGATGGCA | GAACCTTCATTCGCGACAGC |
| F01.PB32155 | *ATP3* | GCTTTGACACTGCCGTTGAG | AAGCAGCAGTCTACATCCCG |
| F01.PB22683 | *SAL2* | CACTCGGTCCGAAAGAGGAG | TGCTTCCGCGGGTATCTTTT |
| F01.PB9432 | *ABR1* | AAATTTTTCCACCGCCACCG | AGCTTTGTGTGGATCGCGTA |
| F01.PB24747 | *CH3.1* | GTCGTGAGGAGAATTGGGCA | GGCTTGAGGTTGAGTCCGAA |
| F01.PB12450 | *LECRK3* | AACGGCAAAGGGAGTCTCAG | CGGCTGATACCACAGAGGAC |
| F01.PB32804 | *LECRK4* | CTGGGAGCTTGAGTATGGGC | ATCCCCTGCCTTGATTCGTG |
| F01.PB17635 | *LECRK7* | TGCGTCTTACAACGCTGGAT | GCCTCTCAGGCTTCTTCACA |
| F01.PB41283 | *U-BOX21* | CACCATCAAAACGTTGCCGT | AGACAGCGGTCTCTTTGAGC |
| F01.PB1298 | *PCL1* | ATTGGAGAGTCTCTGCCCCA | GCGGGTATGTTGGAATTGGC |
| F01.PB27126 | *ERF073* | AACCAGTTTGCGGCATTTCC | GCAACCCCTTCGAGGAGAAA |
| F01.PB27326 | *HDT1* | CGGATGGTGCTCTTTCGTCT | AACCATGAGGACACGACGAC |
| F01.PB46239 | *LRR* | GGCTTGGCAGCCATATCAGA | CACGATGGGTGCTAGTGTCA |
| F01.PB1144 | *LOL1* | GGATCTGAGGCAGTTGGCTT | CTCTGTGCACCATTTGCAGG |
| F01.PB36886 | *AA2* | GCGTGATTCGAATTCGGTGG | TAGTTTCCCAACTCGGCACC |
| Renference | *TUB1* | CAACAAATGTGGGATGCCAA | TGCCATCTTCAGCCCAGTTG |
