# Supplementary material for: Full-length transcriptome and targeted metabolome analyses provide insights into defense mechanisms of Malus sieversii against Agrilus mali
Source: PeerJ. 2020 May 14;8:e8992. doi: 10.7717/peerj.8992 (PMC7231508; doi:10.7717/peerj.8992)
Supplement: Table S2 — Q30(%): the percent of quality value ≥30 in all bases. In this article, Only Clean Data is counted. [file peerj-08-8992-s006.docx]

|  | | | | |
| --- | --- | --- | --- | --- |
| SampleID | ReadSum | BaseSum | GC(%) | Q30(%) |
| T01 | 21,431,374 | 6,414,477,650 | 46.46 | 94.29 |
| T02 | 22,432,825 | 6,692,824,202 | 46.64 | 94.59 |
| T03 | 24,648,881 | 7,376,337,086 | 46.59 | 94.02 |
| T04 | 24,797,780 | 7,419,016,834 | 46.23 | 93.87 |
| T05 | 25,263,266 | 7,561,101,792 | 46.40 | 93.38 |
| T06 | 24,145,087 | 7,224,591,358 | 46.47 | 93.59 |
| T07 | 26,015,392 | 7,782,123,772 | 46.29 | 93.63 |
| T08 | 25,128,427 | 7,507,526,578 | 46.21 | 94.04 |
| T09 | 23,132,624 | 6,927,243,418 | 46.36 | 93.51 |
| T10 | 22,751,767 | 6,815,534,494 | 46.66 | 93.56 |
| T11 | 25,377,792 | 7,601,141,134 | 45.99 | 93.64 |
| T12 | 26,425,665 | 7,910,979,452 | 46.16 | 93.71 |
