# Supplementary material for: Full-length transcriptome and targeted metabolome analyses provide insights into defense mechanisms of Malus sieversii against Agrilus mali
Source: PeerJ. 2020 May 14;8:e8992. doi: 10.7717/peerj.8992 (PMC7231508; doi:10.7717/peerj.8992)
Supplement: Table S3 — Size: the length of insert fragment; Number of consensus isoforms: consensus sequences obtained by ICE clustering; Number of HQ isoforms: number of high-quality (accuracy > 0.99) transcripts in consensus sequence after polish correction; Number of LQ isoforms: Number of low-quality (accuracy < 0.99) transcripts in consensus sequence after polish correction; Percent of HQ isoforms (%): percentage of high-quality transcripts in consensus sequence. [file peerj-08-8992-s007.docx]

|  | | | | | |
| --- | --- | --- | --- | --- | --- |
| Size | Number of consensus isoforms | Average consensus isoforms read length | Number of polished high-quality isoforms | Number of polished low-quality isoforms | Percent of polished high-quality isoforms(%) |
| 0to1kb | 4,717 | 905 | 4,258 | 459 | 90.27% |
| 1to2kb | 31,652 | 1,399 | 27,230 | 4422 | 86.03% |
| 2to3kb | 14,251 | 2,375 | 9,895 | 4356 | 69.43% |
| 3to6kb | 13433 | 3490 | 5797 | 7636 | 43.15% |
| Above6kb | 639 | 9,427 | 1 | 638 | 0.16% |
