# Supplementary material for: Full-length transcriptome and targeted metabolome analyses provide insights into defense mechanisms of Malus sieversii against Agrilus mali
Source: PeerJ. 2020 May 14;8:e8992. doi: 10.7717/peerj.8992 (PMC7231508; doi:10.7717/peerj.8992)
Supplement: Supplemental Information 9 [file peerj-08-8992-s009.docx]

**Response of secondary metabolites after pest infestation in *Malus sieversii***

|  |  | Susceptible | | | Resistant | | |
| --- | --- | --- | --- | --- | --- | --- | --- |
| trans-Cinnamic acid | NON-Feeding | 5880 | 25400 | 45200 | 7120 | 6510 | 59600 |
|  | Feeding | 3830 | 35000 | 59300 | 210000 | 232000 | 94200 |
| Caffeic acid | NON-Feeding | 3890 | 4390 | 3010 | 3660 | 3750 | 6680 |
|  | Feeding | 3350 | 3120 | 5370 | 13600 | 16300 | 9010 |
| p-Coumaric acid | NON-Feeding | 21800 | 14000 | 31500 | 1800 | 5320 | 8320 |
|  | Feeding | 3980 | 16200 | 14700 | 13300 | 11600 | 11500 |
| Vanillic acid | NON-Feeding | 107000 | 133000 | 83000 | 70900 | 151000 | 82300 |
|  | Feeding | 146000 | 83800 | 79100 | 151000 | 145000 | 79700 |
| Syringic acid | NON-Feeding | 117000 | 120000 | 130000 | 32300 | 52700 | 125000 |
|  | Feeding | 47300 | 123000 | 98200 | 96300 | 111000 | 47900 |
| Ferulic acid | NON-Feeding | 23900 | 23600 | 49200 | 49500 | 45500 | 58200 |
|  | Feeding | 46400 | 46400 | 42200 | 52800 | 53100 | 55900 |
| Theophylline | NON-Feeding | 5680 | 11900 | 3940 | 5950 | 2110 | 4480 |
|  | Feeding | 2820 | 3980 | 12300 | 11400 | 6640 | 4660 |
| aloperine | NON-Feeding | 6370 | 2920 | 2610 | 4660 | 2870 | 1920 |
|  | Feeding | 3760 | 1860 | 3620 | 2260 | 1920 | 2090 |
| Theobromine | NON-Feeding | 1150000 | 1120000 | 1150000 | 782000 | 719000 | 1340000 |
|  | Feeding | 739000 | 1130000 | 1040000 | 720000 | 693000 | 742000 |
| Gramine | NON-Feeding | 3410 | 4440 | 3180 | 6140 | 3640 | 4100 |
|  | Feeding | 3780 | 2840 | 3570 | 5110 | 3250 | 4320 |
| Quercetin | NON-Feeding | 21700 | 11300 | 23800 | 18900 | 24100 | 7880 |
|  | Feeding | 24800 | 20600 | 19500 | 12800 | 11300 | 9140 |
| Quercitrin | NON-Feeding | 21200000 | 21400000 | 21700000 | 26700000 | 22600000 | 25800000 |
|  | Feeding | 25100000 | 22500000 | 22800000 | 24300000 | 23900000 | 27400000 |
| Rutin | NON-Feeding | 4750000 | 3720000 | 4600000 | 6620000 | 10300000 | 12500000 |
|  | Feeding | 8220000 | 6550000 | 4920000 | 8380000 | 8990000 | 7180000 |
| Benzoic acid | NON-Feeding | 22500 | 16600 | 19700 | 10600 | 18300 | 41000 |
|  | Feeding | 9400 | 38800 | 26200 | 54200 | 73300 | 9880 |
| Chlorogenic acid | NON-Feeding | 789000 | 2160000 | 12000000 | 890000 | 469000 | 19300000 |
|  | Feeding | 726000 | 8870000 | 9500000 | 22300000 | 24100000 | 19800000 |
| Salicylic acid | NON-Feeding | 840000 | 964000 | 2480000 | 586000 | 784000 | 2520000 |
|  | Feeding | 623000 | 1950000 | 1700000 | 2110000 | 1440000 | 1380000 |

**Number of bugs parasitic on resistance and susceptible strains.**

|  | Number of insects | | | | | | | | | |
| --- | --- | --- | --- | --- | --- | --- | --- | --- | --- | --- |
| Shoots number | 1 | 2 | 3 | 4 | 5 | 6 | 7 | 8 | 9 | 10 |
| Susceptible | 7 | 11 | 5 | 4 | 5 | 7 | 7 | 10 | 6 | 8 |
| Resistant | 2 | 4 | 0 | 2 | 1 | 2 | 1 | 2 | 1 | 1 |

**The average size of infected area in the leaves of resistant and susceptible strains**

| Feeding leaves area (cm2) | | |
| --- | --- | --- |
| Resistant | Susceptible | Feeding days |
| 15 | 78 | 2D |
| 112 | 160 |  |
| 164 | 257 |  |
| 142 | 397 |  |
| 121 | 53 |  |
| 69 | 136 | 4D |
| 118 | 132 |  |
| 109 | 222 |  |
| 5 | 106 |  |
| 61 | 249 |  |
| 2 | 15 | 8D |
| 13 | 29 |  |
| 0 | 34 |  |
| 8 | 46 |  |
| 0 | 20 |  |
| 0 | 108 | 12D |
| 0 | 9 |  |
| 0 | 19 |  |
| 4 | 15 |  |
| 2 | 10 |  |

**Differences in biochemical and infection rates between resistance and susceptible strains.**

| Soluble sugar（mg/g） | | Tannin（mg/g） | | Polyphenols（mg/g） | |
| --- | --- | --- | --- | --- | --- |
| Resistant | Susceptible | Resistant | Susceptible | Resistant | Susceptible |
| 14.83 | 13.65 | 0.63 | 0.15 | 0.08 | 0.05 |
| 11.42 | 18.13 | 0.63 | 0.39 | 0.09 | 0.04 |
| 4.55 | 16.15 | 0.61 | 0.24 | 0.08 | 0.03 |
| 10.18 | 21.68 | 0.61 | 0.49 | 0.06 | 0.07 |
| 8.98 | 21.13 | 0.62 | 0.22 | 0.08 | 0.05 |
| 12.15 | 22.33 | 0.65 | 0.40 | 0.07 | 0.05 |
| 8.50 | 17.32 | 0.54 | 0.26 | 0.08 | 0.07 |
| 3.28 | 17.02 | 0.78 | 0.30 | 0.08 | 0.06 |
| 13.05 | 23.95 | 0.64 | 0.46 | 0.07 | 0.03 |
| 10.87 | 15.42 | 0.56 | 0.23 | 0.07 | 0.07 |
| 14.37 | 17.30 | 0.55 | 0.36 | 0.06 | 0.04 |
| 8.03 | 19.03 | 0.69 | 0.14 | 0.09 | 0.06 |
| 4.22 | 15.70 | 0.67 | 0.29 | 0.07 | 0.03 |
| 12.28 | 16.93 | 0.63 | 0.56 | 0.06 | 0.04 |
| 9.62 | 16.72 | 0.65 | 0.20 | 0.05 | 0.04 |

**Real-time quantitative PCR verification**

| Gene | | Treat | Resistant | | | Susceptible | | |
| --- | --- | --- | --- | --- | --- | --- | --- | --- |
| 1 | TIFY9 | Non-Feeding | 0.017178 | 0.021148 | 0.020713 | 0.043485 | 0.112396 | 0.080029 |
|  |  | Feeding | 0.983957 | 0.856584 | 1.186463 | 1.004632 | 1.04006 | 1.203025 |
| 2 | TCM4 | Non-Feeding | 0.010333 | 0.011787 | 0.011706 | 0.382447 | 0.718636 | 0.579682 |
|  |  | Feeding | 0.802923 | 0.935191 | 1.331759 | 1.295342 | 1.112136 | 1.03766 |
| 3 | BAC7 | Non-Feeding | 0.000173 | 0.0002 | 0.000184 | 0.004776 | 0.010821 | 0.009163 |
|  |  | Feeding | 1.057018 | 0.757858 | 1.248331 | 2.479415 | 2.281527 | 2.584706 |
| 4 | ERF073 | Non-Feeding | 127.5572 | 111.8173 | 161.4563 | 52.89282 | 43.86505 | 48.67132 |
|  |  | Feeding | 0.891929 | 1.121166 | 1.075494 | 8.485502 | 20.32242 | 14.98036 |
| 5 | SAL2 | Non-Feeding | 0.001874 | 0.003023 | 0.003044 | 0.236514 | 0.594604 | 0.566442 |
|  |  | Feeding | 0.926588 | 0.852635 | 1.265757 | 1.777685 | 1.70527 | 1.866066 |
| 6 | ABR1 | Non-Feeding | 0.000577 | 0.000725 | 0.000886 | 0.00982 | 0.015843 | 0.010672 |
|  |  | Feeding | 0.946058 | 0.90125 | 1.172835 | 0.888843 | 0.852635 | 0.926588 |
| 7 | LECRK3 | Non-Feeding | 0.004082 | 0.003385 | 0.005275 | 0.073472 | 0.221186 | 0.193893 |
|  |  | Feeding | 1.023374 | 0.814131 | 1.200249 | 1.104454 | 1.331759 | 1.159364 |
| 8 | LECRK4 | Non-Feeding | 0.03176 | 0.031106 | 0.034039 | 0.050883 | 0.039922 | 0.058449 |
|  |  | Feeding | 1.059463 | 0.814131 | 1.159364 | 0.302149 | 0.240371 | 0.247129 |
| 9 | PCL1 | Non-Feeding | 0.000303 | 0.00032 | 0.000338 | 0.038473 | 0.086569 | 0.082469 |
|  |  | Feeding | 0.939523 | 0.870551 | 1.22264 | 0.632878 | 0.547147 | 0.607097 |
| 10 | U-BOX21 | Non-Feeding | 0.0000764 | 0.000174 | 0.000148 | 0.005934 | 0.008569 | 0.009312 |
|  |  | Feeding | 1.059463 | 0.819794 | 1.151355 | 0.263036 | 0.227405 | 0.17115 |
| 11 | ATP3 | Non-Feeding | 0.001353 | 0.00588 | 0.001069 | 0.018453 | 0.050415 | 0.040107 |
|  |  | Feeding | 0.939523 | 0.846745 | 1.257013 | 0.435275 | 0.346277 | 0.389582 |
| 12 | CH3.1 | Non-Feeding | 0.000102 | 0.000272 | 0.000245 | 0.003369 | 0.010501 | 0.006244 |
|  |  | Feeding | 0.943874 | 0.850667 | 1.24545 | 0.177595 | 0.165702 | 0.180075 |
| 13 | LECRK7 | Non-Feeding | 0.000307 | 0.000345 | 0.000399 | 0.00588 | 0.017824 | 0.01038 |
|  |  | Feeding | 0.946058 | 0.823591 | 1.283426 | 1.231144 | 1.049717 | 1.292353 |
| 14 | EDR1 | Non-Feeding | 0.004776 | 0.005226 | 0.00564 | 0.568567 | 0.933033 | 0.835088 |
|  |  | Feeding | 0.986233 | 0.852635 | 1.189207 | 1.22688 | 1.180993 | 1.375542 |
| 15 | HDT1 | Non-Feeding | 0.037944 | 0.044502 | 0.047039 | 2.094294 | 2.496661 | 2.158456 |
|  |  | Feeding | 0.993092 | 0.835088 | 1.205808 | 1.257013 | 1.148698 | 1.337928 |
| 16 | LRR | Non-Feeding | 0.183434 | 0.177186 | 0.213652 | 5.327037 | 8.018505 | 7.638733 |
|  |  | Feeding | 1.200249 | 0.749154 | 1.112136 | 1.487958 | 1.268684 | 1.54043 |
| 17 | LOL1 | Non-Feeding | 0.128812 | 0.118531 | 0.119355 | 18.475705 | 18.67884 | 17.5492 |
|  |  | Feeding | 0.948246 | 0.909618 | 1.159364 | 10.65407 | 8.958973 | 10.9536 |
| 18 | AA2 | Non-Feeding | 0.059954 | 0.081334 | 0.075363 | 43.58831 | 53.44563 | 45.25483 |
|  |  | Feeding | 0.913831 | 0.895025 | 1.22264 | 10.55606 | 9.38268 | 10.55606 |
